# Neuroendocrine tumours and their microenvironment

**DOI:** 10.1007/s00262-020-02556-1

**Published:** 2020-04-08

**Authors:** Lotte D. de Hosson, Tim J. Takkenkamp, Gursah Kats-Ugurlu, Grietje Bouma, Marian Bulthuis, Elisabeth G. E. de Vries, Martijn van Faassen, Ido P. Kema, Annemiek M. E. Walenkamp

**Affiliations:** 1 Department of Medical Oncology, University Medical Centre Groningen, University of Groningen, DA11, PO Box 30.001, 9700 RB Groningen, The Netherlands; 2 Department of Pathology, University Medical Centre Groningen, University of Groningen, Groningen, The Netherlands; 3 Department of Laboratory Medicine, University Medical Centre Groningen, University of Groningen, Groningen, The Netherlands

**Keywords:** Neuroendocrine tumours, Programmed death-ligand-1, Indoleamine 2,3-dioxygenase, Tryptophan 2,3-dioxygenase, T-cells, Immune microenvironment

## Abstract

Tumours can escape the immune system by expressing programmed death-ligand-1 (PD-L1), which allows them to bind to PD-1 on T-cells and avoid recognition by the immune system. Regulatory T-cells (Tregs), indoleamine 2,3-dioxygenase (IDO) and tryptophan 2,3-dioxygenase (TDO) also play a role in immune suppression. Knowledge about the interaction of neuroendocrine tumours (NETs) with their immune microenvironment and the role of immunotherapy in patients with NET is scarce. Here, we investigated the immune microenvironment of serotonin-producing (SP) and non-serotonin-producing NETs (NSP-NETs). Tumours of 33 patients with SP-NET and 18 patients with NSP-NET were studied. Immunohistochemical analyses were performed for PD-L1, T-cells, IDO, TDO, mismatch repair proteins (MMRp) and activated fibroblasts. PD-L1 expression was seen in < 1% of tumour and T-cells. T-cells were present in 33% of NETs, varying between 1 and 10% T-cells per high power field. IDO was expressed in tumour cells in 55% of SP-NETs and 22% of NSP-NETs (*p* = 0.039). TDO was expressed in stromal cells in 64% of SP-NETs and 13% of NSP-NETs (*p* = 0.001). No tumours had loss of MMRp. TDO-expressing stromal cells also strongly expressed α-SMA and were identified as cancer-associated fibroblasts (CAFs). Factors that are associated with a response to checkpoint inhibitor treatment were absent or only present to a limited extent in the tumour microenvironment of NETs. The expression of IDO and TDO in a substantial part of NETs and the presence of CAFs suggest two mechanisms that could be responsible for the cold immune microenvironment, which should be explored to enhance anti-tumour immunity and clinical responses.

**Electronic supplementary material:**

The online version of this article (10.1007/s00262-020-02556-1) contains supplementary material, which is available to authorized users.

## Introduction

Neuroendocrine tumours (NETs) comprise a heterogeneous group of tumours that are predominantly derived from the enterochromaffin cells of the gastro-enteropancreatic tract or bronchopulmonary system [2]. NETs grade 1 and 2 can produce various biogenic amines and polypeptide hormones, of which serotonin is the most common [3]. Radical resection of the NET is the only curative possibility. However, NET patients often present with non-resectable or metastatic disease. Non-curative systemic treatment aimed at controlling symptoms and progression of disease includes somatostatin analogues, interferon, everolimus, sunitinib, peptide receptor radionuclide therapy and chemotherapy [4]. None of the currently used systemic treatments for NET grade 1 and 2 were rated as having substantial clinical benefit according to the ‘European Society of Medical Oncology magnitude of clinical benefit scale’ [5]. Consequently, there is an unmet need for new and better systemic treatment modalities.

In recent last years, immunotherapy with immune checkpoint inhibiting antibodies has shown anti-tumour activity across numerous tumour types. These therapies target the cell surface proteins programmed death-ligand-1 (PD-L1) on tumour and immune/non-immune cells and programmed death-1 (PD-1) on monocytes/lymphocytes [6]. Targeting PD-L1 and PD-1 leads to activated T-cells in the tumour microenvironment. However, little research has been published on the activity of these drugs in NETs.

Clinical studies have shown that a number of additional factors are associated with a response to checkpoint inhibitor treatment, such as the presence of T-cells and high mutational tumour load [7]. In addition, major interest has been shown in the tryptophan-degrading enzymes indoleamine 2,3-dioxygenase (IDO) and tryptophan 2,3-dioxygenase (TDO). These enzymes convert tryptophan into kynurenine, along the kynurenine pathway, which could lead to depletion of tryptophan in the tumour microenvironment [8–10]. Tryptophan is also the precursor of serotonin. IDO and TDO are especially of interest in NETs as these tumours often produce serotonin, which potentially depletes its precursor tryptophan [11].

Surprisingly little is currently known about the complex interactions of NET cells with their surrounding immune microenvironment. Studies examining T-cells in neuroendocrine neoplasms showed the presence of T-cells in most NETs, but the amount of T-cells varied and number of tumours with Tregs or FoxP3-positive cells varied between 26 and 34% [12, 13]. Furthermore, limited PD-L1 expression in NET cells has been observed in six studies, while in two other studies more than half of NETs had PD-L1 expression [14–16]. The use of different PD-L1 antibodies in the various studies may account for these heterogeneous findings.

We investigated the tumour immune microenvironment—in particular the presence of PD-L1, T-cells, IDO, TDO, MMRp and cancer-associated fibroblasts (CAFs)—in tissue from patients with treatment-naive serotonin-producing (SP) and non-serotonin-producing NET (NSP-NET) grade 1 or 2 in this exploratory study. Furthermore, we related the immunohistochemical data to overall survival of the patients.

## Material and methods

### Participants

Medical records of newly referred NET patients to the Department of Medical Oncology of the University Medical Center Groningen (UMCG) between January 1, 2008, and December 31, 2014, were screened. Patients diagnosed with a NET grade 1 or 2 according to the World Health Organization 2010 classification were selected. A patient was diagnosed with a SP-NET when urinary 24-h excretion of 5-HIAA was > 3.8 mmol/mol creatinine and/or platelet serotonin > 5.4 nmol/10^9^ platelets [10]. Serotonin production and 5-hydroxyindolacetic acid (5-HIAA) in 24-h (24-h) urine were measured by high-performance liquid chromatography (HPLC) [17].

We included NET patients with tumour tissue, platelet-rich plasma (PRP) for analysis of serotonin and/or urinary 24-h excretion of 5-hydroxyindolacetic acid (5-HIAA) available before start of systemic anti-tumour treatment. Somatostatin analogue use was allowed for maximal 14 days before tumour tissue was collected. We excluded NET patients with other primary solid/haematological malignancy, auto-immune disease (e.g. colitis) or infectious disease (e.g. hepatitis) as well as patients with concomitant use of treatment interfering with IDO activity (e.g. interferon).

Patients were clinically staged according to the Union for International Cancer Control guidelines [18]. Histopathological analysis of the formalin-fixed paraffin-embedded tumour tissue of the patients was centrally reviewed by a dedicated NET pathologist (Gursah Kats-Ugurlu).

### Tumour histology and Immunohistochemistry

In all tumour samples, 3-µm slides of formalin-fixed paraffin-embedded tumour samples were studied for morphology and mitotic count on standard hematoxylin and eosin (HE) stain, and proliferation index was determined using immunohistochemistry-based Ki-67 stain (mouse anti-Ki67, MiB-1 clone, dilution 1:100 Dako, Glostrup, Denmark). Two antibodies for PD-L1 staining were used: mouse anti-PD-L1 (clone 22C3, 1:50, Dako, Glostrup, Denmark) and rabbit anti-PD-L1 (clone E1L3N, 1:200 Cell Signaling Technology, Danvers, MA, USA). PD-L1 antibodies were applied in the Ventana Ultra staining system. To detect MMR antigens, we used anti-MLH-1 mouse monoclonal primary antibody (clone M1, Roche Diagnostics, IN, USA), PMS2 rabbit monoclonal antibody (clone EPR3947, Cell Marque, CA, USA), MSH2 mouse monoclonal antibody (clone G219-1129, Cell Marque) and CONFIRM anti-MSH6 mouse monoclonal primary antibody (clone 44, Roche Diagnostics). The mouse anti-CD3 antibody (1:50, Monosan, Sanbio, Uden, the Netherlands) was used to recognise T-cells. Antibodies against IDO (mouse anti-IDO, MAB5412, 1:25, Chemicon, Millipore, Amsterdam, the Netherlands), TDO (rabbit anti-TDO2, clone HPA 039,611, 1:200, Atlas Antibodies, Bromma, Sweden) desmin (M0760, dilution Dako 1:50, Madrid, Spain) and α-SMA (mouse anti-SMA, clone 1A4, 1:1000, Sigma, MI, USA) were applied. α-SMA and desmin are well-established markers for myofibroblasts and myofibroblast-like cells in the tumour microenvironment, also known as CAFs [19, 20].

One pathologist (Gursah Kats-Ugurlu) and two researchers (Grietje Bouma and Lotte D. de Hosson) evaluated the stained slides at a double-head microscope blinded for clinical and histopathological information. To avoid a learning effect, every first 10 slides that were scored and slides that were difficult to score were scored again without knowledge of the first score. Positive external controls were placenta for PD-L1, appendix for the MMR antigens and CD3, lymph node for IDO and prostate for TDO. For α-SMA, the pericytes of blood vessels served as internal control.

The tumour and its immediate environment were evaluated. Tumour cells with ≥ 1% positive staining for PD-L1, IDO and TDO were defined as positive. MMRp loss was assumed if tumour cell nucleus showed no staining in comparison with internal inflammatory cells or appendix as external control. T-cells were scored ‘present’ if ≥ 1% of the cells in a high power field composed of T-cells distributed patchily or diffusely in CD3. PD-L1 showed a membranous staining pattern of tumour cells, and IDO showed a cytoplasmic staining pattern with the presence of acellular small depositions. Besides a cytoplasmic staining pattern, TDO showed expression in tumour stroma. α-SMA staining was performed to further characterise TDO-positive stromal cells on five tumours with TDO expression in stroma and on five without TDO expression in stroma. Furthermore, staining with desmin, another marker for CAFs, was performed. Virtual double staining (VDS) (Visiopharm©) with HE and α-SMA showed the close vicinity of stroma cells that were identified as CAFs and tumour cells. Stained slides were scanned at an objective magnification of 40× using Philips Ultra Fast Scanner 1.6 and were saved in the image file format i-Syntax.

The method described below was used for the analysis of the slides. First, alignment on a large scale was performed on the images of adjacent slides stained for HE and α-SMA. Visiopharm presented the best possible match of the 2 tissue sections on a finer detail level, which was verified by a technician. Thereafter, cells were identified by shape and size with a fully automated watershed-based segmentation technique to separate cells positive and negative for α-SMA from the background. Double staining for α-SMA and TDO was performed using an alkaline phosphatase-based blue chromogen for α-SMA and using nova-red chromogen for TDO. To determine which T-cell subsets were present, VDS was used for 3 tumours stained with CD3 and CD8 antibodies (ready to use antibodies of Ventana BenchMark). The CD3-positive and CD8-positive cells were counted manually by the pathologist, and the CD8/CD3 ratio was calculated.

### Overall survival

Overall survival data were obtained from patient records in December 2019.

### Statistical analysis

For this exploratory study, no sample size calculation was performed. Descriptive statistics (e.g. median, ranges and frequencies) were calculated for all measures. The Mann–Whitney U test was used to compare distributions across groups. Associations of categorical variables were tested using the Chi-square test. Hazard ratios for relation between immunohistochemical data and overall survival were calculated with Cox regression analysis.

Tests were performed two-sided, and *p* values < 0.05 were considered significant. Analyses were executed using the software package SPSS, version 23 for Windows (SPSS, Inc, Chicago, IL, USA).

## Results

### Patient characteristics

Clinical and pathological characteristics at the time of tumour collection of all SP-NET (*N* = 33) and NSP-NET (*N* = 18) patients are summarised in Table 1. Thirteen SP-NET patients were treated briefly with somatostatin analogues before tissue sampling. The brief use of somatostatin analogues was unavoidable in 13 out of 51 (25%) patients, as this was prescribed to prevent a carcinoid crisis during an invasive procedure.

**Table 1:** Clinicopathological characteristics of patients

	NET patients (*N* = 51) ^a^	NSP NET (*N* = 18)	SP NET (*N* = 33)
Mean age in years ± SD	62.8 ± 11.0		
Male sex	30 (59)	11 (61)	19 (58)
*Primary tumour location*			
Lung	2 (4)	2 (11)	0 (0)
Stomach	1 (2)	1 (6)	0 (0)
Duodenum	2 (4)	2 (11)	0 (0)
Pancreas	14 (27)	11 (61)	3 (9)
Jejunum/ileum	22 (43)	0 (0)	22 (67)
Colon/rectum	2 (4)	1 (6)	1 (3)
Unknown	8 (16)	1 (6)	7 (21)
*Tumour grade*			
Grade 1	34 (67)	8 (44)	25 (76)
Grade 2	16 (31)	10 (56)	6 (18)
Unknown	1 (2)	0 (0)	2 (6)
*Disease stage*			
Stage 1/2	3 (6)	2 (11)	1 (3)
Stage 3/4	46 (90)	15 (83)	31 (94)
Unknown	2 (4)	1 (6)	1 (3)
*Source of tissue sample*			
Primary tumour	30 (59)	10 (56)	20 (61)
Metastasis	20 (39)	8 (44)	12 (36)
Unknown	1 (2)	0 (0)	1 (3)
*Location of tissue sample*			
Liver	14 (27)	8 (44)	6 (18)
Lymph node	4 (8)	1 (6)	3 (9)
Jejunum/ileum	18 (35)	0 (0)	18 (55)
Pancreas	5 (10)	3 (17)	2 (6)
Duodenum	3 (6)	3 (17)	0 (0)
Lung	2 (4)	1 (6)	1 (3)
Other^b^	5 (10)	2 (6)	3 (9)

*NET* neuroendocrine tumour, *NSP-NET* non-serotonin-producing neuroendocrine tumour, *SP-NET* serotonin-producing neuroendocrine tumour

^a^Values are reported as number (percentage) unless noted otherwise

^b^Other sites of the tissue sample collection were mesenterium of the small intestine (*N* = 2), colon (*N* = 1), stomach (*N* = 1), peritoneum (*N* = 1)

### Expression of PD-L1, MMR, the presence of tumour-infiltrating T-cells, IDO and TDO in NETs

**Fig. 1:**
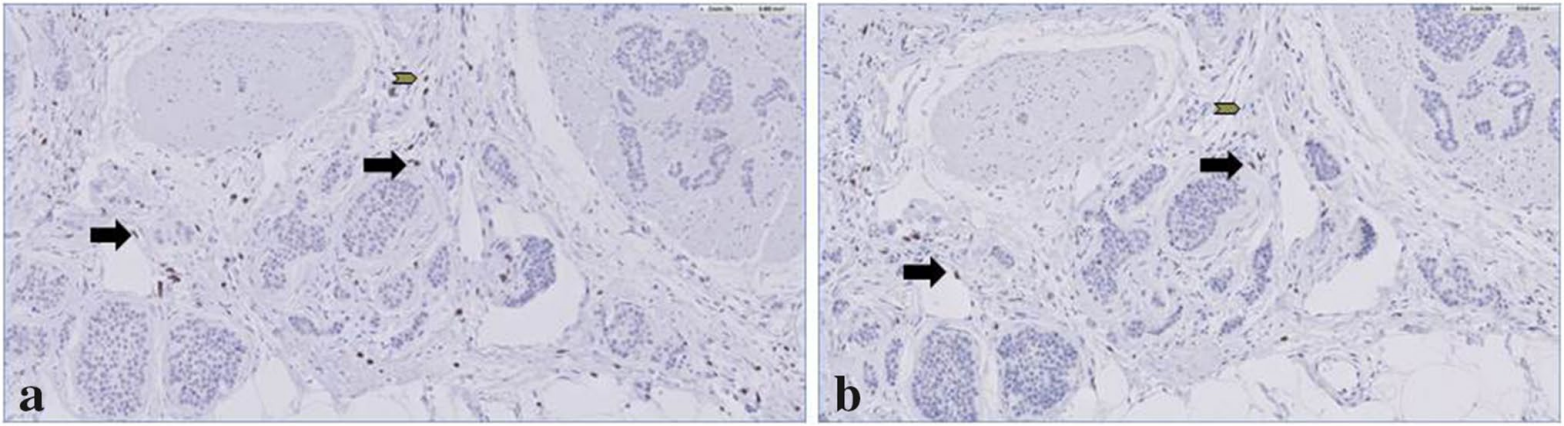
The presence of CD3 and CD8 expressing T-cells in a serotonin-producing NET of the ileum. CD3 and CD8 expressing T-cells in a serotonin-producing ileum resection specimen. **a** CD3 expression (200×), **b** CD8 expression (200×). Cells presenting both CD3 and CD8 (black arrows) and other cells that are only CD3 positive (grey arrows)

None of the tumours were positive for PD-L1 when stained with anti-22C3 or anti-E1L3N stains. None of the tumours showed loss of the MMRp, and T-cells were present in 15 of the 45 samples, varying between 1 and 10% T-cells per high power field (Table 2). T-cells were most frequently found within the stroma of NETs of the jejunum/ileum (in 7 of 22 samples), which were all SP-NETs (Table 3). Of 3 tumours that presented T-cells, the CD8/CD3 ratio was 70/120, 6/25 and 3/10, respectively (Fig. 1). Correlation between T cell infiltration versus MMRp, IDO, TDO could not be detected due to small numbers in the subgroups.

**Table 2:** The presence of T-cells, expression of IDO, TDO, PD-L1 and MMRp in NETs

	All NET *N* (%)	NSP-NET *N* (%)	SP-NET *N* (%)	*p*-value
Tissue samples	51 (100)	18 (100)	33 (100)	
*PD-L1 expression (22C3 antigen)*				
Negative	51 (100)	18 (100)	33 (100)	NS
Positive	0 (0)	0 (0)	0 (0)	
*PD-L1 expression ( E1L3N antigen)*				
Negative	51 (100)	18 (100)	33 (100)	NS
Positive	0 (0)	0 (0)	0 (0)	
*MMRp*				
Loss of MMRp	0 (0)	0 (0)	0(0)	NS
No loss of MMRp	51 (100)	18 (100)	33 (100)	
*T-cells*				
Absent	30 (59)	10 (56)	20 (61)	NS
Present	15 (29)	6 (33)	9 (27)	
Not evaluable	6 (12)	2 (11)	4 (12)	
*IDO expression*				
Negative	29 (57)	14 (78)	15 (45)	0.039
Positive	22 (43)	4 (22)	18 (55)	
*TDO expression in tumour cells*				
Negative	29 (63)	11 (65)	18 (62)^a^	NS
Positive	17 (37)	6 (35)	11 (38)	
Not evaluable	5 (10)	1 (6)	4 (12)	
*TDO expression in stroma*				
Negative	23 (56)	14 (88)	9 (36)	0.001
Positive	18 (44)	2 (13)	16 (64)	
Not evaluable	10 (20)	2 (11)	8 (24)	

*IDO* indoleamine 2,3-dioxygenase, *MMRp* mismatch repair proteins, *NET* neuroendocrine tumour, *NSP-NETs* non-serotonin-producing neuroendocrine tumour, *PD-L1* programmed death-ligand 1, *SP-NETs* serotonin-producing neuroendocrine tumour, *TDO* tryptophan 2,3-dioxygenase

^a^Three tissue samples of SP-NETs with not evaluable stroma were TDO positive in the tumour

**Table 3:** The presence of T-cells and immunohistochemical expression of IDO, TDO and PD-L1 classified by primary origin of the NET

	Jejunum/ileum *N*	Pancreas *N*	Lung *N*	Unknown *N*	Other *N*^a^
Tissue samples	22	14	2	8	5
*T-cells*					
Absent	13	11	0	5	1
Present	7	1	1	3	3
Not evaluable	2	2	1	0	1
*IDO expression*					
Negative	9	9	2	6	3
Positive	13	5	0	2	2
*TDO expression in tumour cells*					
Negative	15	6	2	3	3
Positive	4	6	0	5	2
Not evaluable	3	2	0	0	0
*TDO expression in stroma*					
Negative	2	12	1	5	3
Positive	15	1	0	1	1
Not evaluable	5	1	1	2	1
PD-L1 expression	0	0	0	0	0
MMRp	0	0	0	0	0

*IDO* indoleamine 2,3-dioxygenase, *MMRp* mismatch repair proteins, *NET* neuroendocrine tumour, *PD-L1* programmed death-ligand 1, *TDO* tryptophan 2,3-dioxygenase

^a^Other sites of primary origin of the NET are stomach (*N* = 1), duodenum (*N* = 2), colon (*N* = 1), rectum (*N* = 1)

IDO expression was restricted to tumour cells and varied between focal and diffuse presence of intracytoplasmic acellular small depositions. IDO expression in tumour cells was more frequently observed in SP-NETs (55%, 18/33) than in NSP-NETs (22%, 4/18) (*p* = 0.0039, Table 2).

**Fig. 2:**
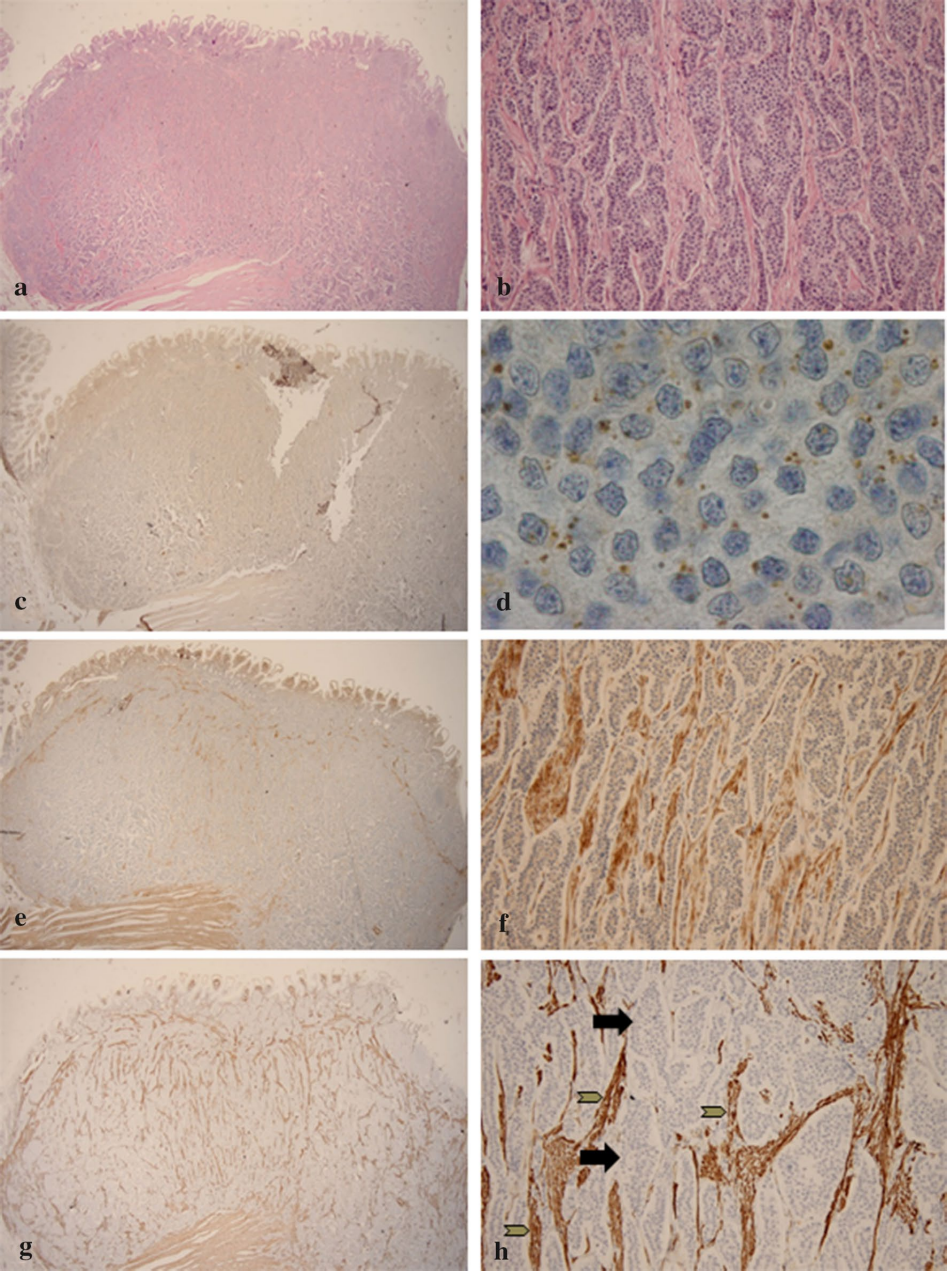
Expression of IDO, TDO and α-SMA and IDO in a serotonin-producing NET of the ileum. Illustrative images of indoleamine 2,3-dioxygenase (IDO) and tryptophan 2,3-dioxygenase (TDO) expression in a serotonin-producing neuroendocrine tumour (NET) in ileum resection specimen. (**a** HE, 20×). Magnification of the submucosal NET (**b** HE, 200×). At lower magnification, IDO is not detectable (**c**, IDO, 20×) In higher magnification, a diffuse, strong brown intracytoplasmic, dot-like IDO expression (marked by arrows) is seen in tumour cells (**d** IDO, 1000×). TDO expression is visible in stromal cells surrounding the tumour cells (**e** TDO, 20× , **f** TDO, 200×). α-SMA is expressed in stromal cells (grey arrows) between tumour cells (black arrows) and has a stronger expression in areas with more TDO expression (**g** α-SMA, 20×, **h** α-SMA, 200×)

**Fig. 3:**
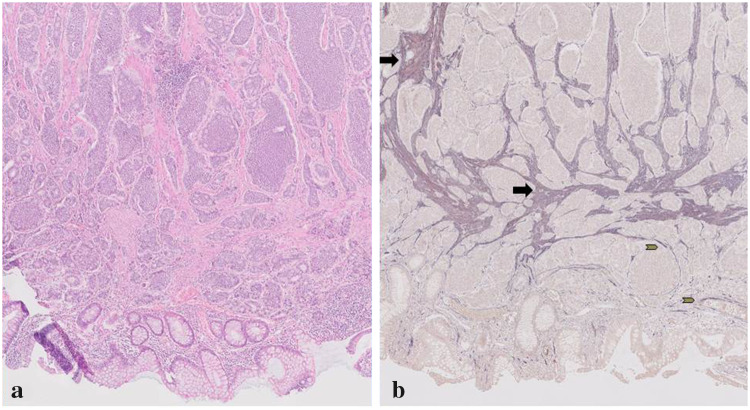
Double staining in a serotonin-producing NET of the ileum. Illustrative image of double staining with α-SMA and TDO expression in a serotonin-producing ileum resection specimen. **a** HE staining (50×), **b** α-SMA in alkaline phosphatase (blue) and TDO expression in nova-red (red) (50×). Purple colour shows stromal cells with expression of α-SMA and TDO (black arrows), and others only express α-SMA (grey arrows)

**Fig. 4:**
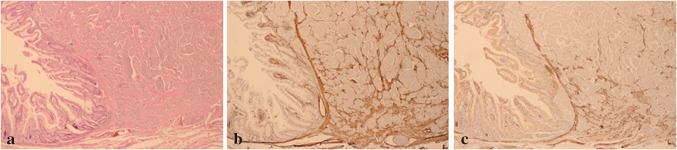
Expression of α-SMA and desmin in a serotonin-producing NET of the ileum. Illustrative images of α-SMA and desmin expression in a serotonin-producing ileum resection specimen. **a** (HE 400×), **b** (α-SMA 400×), **c** (desmin 400×)

Three distinct patterns of TDO expression were found in the NETs: in tumour cells, in stroma or in both (Fig. 2). NETs expressed TDO in either the tumour cells (37%, 17/46) or stroma (44%, 18/41) (Table 2). Remarkably, TDO in stroma was observed in 64% (16/25) of evaluable SP-NETs and 13% (2/16) of the NSP-NETs (*p* = 0.0001). To investigate the origin of these TDO-positive stromal cells, α-SMA staining was performed on 10 slides, which showed that these cells strongly expressed α-SMA and desmin and were spindle-shaped. This was confirmed with double staining of α-SMA and TDO (Fig. 3). Furthermore, these cells were located within the vicinity of tumour cells, as shown with VDS (Fig. 4). The stromal cells were therefore identified as CAFs [21–23].

### Relation of immunohistochemical data and overall survival

Median follow-up was 63 months (range 1–162 months); 26 patients died. No statistical significant differences in overall survival were seen in patients with the presence or absence of either CD3, IDO or TDO in tumour, nor for TDO in stroma (Supplementary data).

## Discussion

Our exploration of the immune microenvironment of NETs revealed that tumour cells in NETs did not express PD-L1. In addition, only a minority of NETs contained a small number of T-cells. A substantial proportion of NETs expressed IDO and TDO, and TDO was also expressed by stromal cells. None of the tumours were MMRp-deficient. These cells were identified as CAFs due to the positive staining with α-SMA and desmin. Furthermore, VDS showed their close vicinity to tumour cells [19, 20].

Our study showed that NETs exhibit a ‘cold’ tumour microenvironment lacking several characteristics that have recently been associated with a response to checkpoint inhibitor treatment [6]. The expression of IDO and TDO in a substantial proportion of NETs and the presence of CAFs suggest that these two mechanisms could be responsible for the cold immune microenvironment.

Interestingly, an immunohistochemical analysis of lung cancer specimens revealed increased TDO2 expression in CAFs. Furthermore, administration of TDO2 inhibitor improved T-cell response and decreased tumour metastasis in mice with metastatic lung cancer [24]. The enzymes IDO and TDO degrade tryptophan into kynurenines, resulting in a tryptophan depleted tumour microenvironment rich of kynurenines. This microenvironment leads to suppression of effector T-cells or the conversion to tumour tolerant regulatory T-cells (Tregs) [8–10]. A number of IDO and TDO inhibitors have advanced into clinical trials with or without PD-1 antibody inhibitors in patients with solid tumours. Also an IDO1/TDO dual inhibitor is currently being tested in a phase I study [25, 26]. In the ECHO-301/KEYNOTE 252 study, patients with unresectable or metastatic melanoma were randomised to receive pembrolizumab in combination with either epacadostat (an IDO-inhibitor) or placebo. Unfortunately, this phase III study was recently terminated early due to failure to improve progression-free survival (PFS) [27].

Preclinical studies have also suggested that aryl hydrocarbon receptor could become another possible target of the IDO/TDO kynurenine pathway in the treatment of cancer, due to its immunosuppressive role in tumours [28]. Our data showed that especially serotonin-producing NET tumours express IDO and TDO. These patients often have low tryptophan levels, as tryptophan is the sole precursor of peripherally and centrally produced serotonin. This suggests that IDO-mediated immune suppression is most prominent in patients with low tryptophan levels, and these patients might therefore be interesting candidates for treatment with immune checkpoint inhibitors combined with IDO inhibitors. In our study, however, we did not find an association between IDO expression and the presence of T-cells, which might be an indication of the complex interaction of tumours and their immune microenvironment.

Preclinical studies in mouse models of various cancer types showed that CAFs, together with other stromal cells, are responsible for restricting the accumulation of T-cells in the vicinity of cancer cells [29]. Furthermore, CAFs act by secreting various growth factors such as transforming growth factor-beta (TGF-β) [22, 30]. Increased TGF-β in the tumour immune microenvironment was recently shown to represent a primary mechanism of immune evasion that promotes T-cell exclusion [31]. In patients with metastatic urothelial carcinoma, lack of response to immune checkpoint blockade is associated with increased TGF-β signalling in fibroblasts in the tumour microenvironment [31]. Combining TGF-β blockade with immune checkpoint blockade in mouse models increases the anti-tumour efficacy of the therapy, suggesting that identifying and targeting microenvironmental regulators of anti-tumour immunity may increase the reach of immunotherapeutical approaches [32].

Therefore, we used two validated PD-L1 antibodies—22C3 and E1L3N—which both showed no expression of PD-L1 in NET cells.

In contrast to NETs, grade 3 neuroendocrine neoplasms (NENs) were characterised by strong PD-L1 expression [33]. A correlation between tumour grade and PD-L1 expression was also seen in neuroendocrine neoplasms of pulmonary origin [15, 34]. One study involving 32 patients treated with systemic therapy for gastro-intestinal and pancreatic NENs found that PD-L1 expression was associated with PFS [35]. Furthermore, a multivariate analysis of 80 pulmonary NENs revealed that PD-L1 expression, PD1 expression and distant metastasis of pulmonary NENs were independently associated with survival time [15].

In the current study, we observed a paucity of T-cells with fewer CD8-positive cells, in line with several other studies analysing NET immunohistochemically [36]. In one study, samples from 31 midgut NETs (grade 1 and 2) were analysed. Overall, the tumours contained a higher proportion of Tregs compared with matched normal tissue [36]. Other studies examining T-cells in neuroendocrine neoplasms reported more CD3 expressing T-cells. In one of these studies, 68% of pancreatic NETs (*N* = 87) and 97% of NET liver metastases of patients (*N* = 39) with various primary tumour sites contained > 10 intratumoural T-cells per 10 high power fields. Tregs were found in 34% of pancreatic NETs and 33% of NET liver metastases [12]. Other studies reported found tumour-infiltrating lymphocytes (TILs) in 14 out of 17 samples, varying from single positive cells to multiple positive cells [13].

In our study, the paucity of T-cells in NETs and the relatively high rate of Tregs might be the result of CAFs and their secreted factors and of IDO and TDO expression in the tumour and microenvironmental cells. In other tumour types, such as colorectal, oesophageal and endometrial cancer, an inverse correlation between IDO expression and T-cells has been observed [37]. We could not demonstrate a difference in the presence of T-cells in SP-NET and NSP-NET samples although serotonin is considered a pro-inflammatory modulator that promotes invasion of neutrophils and other cells in acute inflammation. Further studies in larger sample sizes are therefore needed [38, 39].

The PD-1 antibodies pembrolizumab and nivolumab are registered for the treatment of several tumour types. Furthermore, nivolumab is registered for MMRp-deficient metastatic colorectal cancer and pembrolizumab for MMRp-deficient tumours, irrespective of the primary tumour [7]. In MMRp-proficient tumours, probable DNA base pairing errors are corrected in newly replicated DNA, leading to microsatellite stable tumours. The equivalence of microsatellite instability testing and MMR immunohistochemistry was shown in endometrium carcinoma [40]. The validity of immunohistochemistry for MMRp to identify patients with microsatellite instable cancer has also been shown for colorectal cancer [41]. In our study, all investigated NETs were MMRp-proficient. This is in line with a study of 35 pancreatic NETs and 34 small intestinal NETs in which all pNETs and 31 small intestinal NETs were MMRp-proficient, and with another study in which all included 70 pancreatic NETs were microsatellite-stable [14].

Little data have been published on treatment with immune checkpoint inhibition in NET patients. In the multicohort phase 1B KEYNOTE-028 study, 41 pre-treated patients with pancreas, lung and gut NET were treated with pembrolizumab. Results showed four patients with objective response and 29 patients with stable disease. Eight patients had severe (grade ≥ 3) treatment-related adverse events [42]. In the phase 2 KEYNOTE-158 study, 107 patients with well-differentiated and moderately differentiated NETs of several primary sites were treated with pembrolizumab [43]. Four patients had a partial response, all patients with a PD-L1 negative tumour. Sixty-one patients had stable disease, and the remaining had progressive disease. In a phase II trial, 33 patients with low-grade, intermediate-grade and high-grade NETs were treated with a dual checkpoint blockade nivolumab and ipilimumab. Partial or complete responses were seen only in patients with high-grade NETs: overall response rate of this group was 44% [44]. An explanation for the better response in high-grade NETs may be the presence of a higher tumour mutational burden.

Multiple phase 1 and 2 trials are currently recruiting NET patients for immune checkpoint inhibitor treatment [45].

Our findings indicate that both tumour and CAFs express IDO and TDO in NET patients. We therefore hypothesise that NET patients will potentially benefit from combination immunotherapies to overcome this cold immune microenvironment. Further studies could elucidate the complex immune microenvironment of NETs.

## Electronic supplementary material

Below is the link to the electronic supplementary material.

Supplementary file1 (DOCX 12 kb)

## Abbreviations

5-HIAA: 5-Hydroxyindolacetic acid
α-SMA: α-Smooth muscle antigen
CAF: Cancer associated fibroblast
HE: Hematoxylin and eosin
HPLC: High-performance liquid chromatography
IDO: Indoleamine 2,3-dioxygenase
MMRp: Mismatch repair proteins
NEN: Neuroendocrine neoplasm
NET: Neuroendocrine tumour
NSP-NET: Non-serotonin-producing NET
PD-L1: Programmed death-ligand-1
PD-1: Programmed death-1
PFS: Progression-free survival
PRP: Platelet-rich plasma
SP-NET: Serotonin-producing NET
TGF-β: Transforming growth factor-beta
TDO: Tryptophan 2,3-dioxygenase
Treg: Regulatory T-cell
VDS: Virtual double staining
