# Supplementary material for: Neuroendocrine tumours and their microenvironment
Source: Cancer Immunol Immunother. 2020 Apr 8;69(8):1449–59. doi: 10.1007/s00262-020-02556-1 (PMC7347684; doi:10.1007/s00262-020-02556-1)
Supplement: Supplementary file 1 — Supplementary file1 (DOCX 12 kb) [file 262_2020_2556_MOESM1_ESM.docx]

**Supplementary data**

**Table S1. Relation of presence of T-cells, immunohistochemical expression of IDO, TDO in tumor and TDO in stroma related with overall survival.**

| Immunohistochemistical staining | Hazard ratio for overall survival  (95% confidence interval) |
| --- | --- |
| T-cells | 2,1 (0,8-5,7) p=0,15 |
| IDO expression | 0,6 (0,2- 1,8) p=0,37 |
| TDO expression in cytoplasma | 1,6 (0,5-5,0) p=0,43 |
| TDO expression in stroma | 0,9 (0,3-2,7) p=0,90 |

CI95: 95% confidence interval IDO: indoleamine 2,3-dioxygenase; TDO: tryptophan 2,3-dioxygenase.
